# Optimizing pre-conception care in South Asia

**DOI:** 10.1016/j.lansea.2025.100586

**Published:** 2025-04-24

**Authors:** Zulfiqar A. Bhutta, Nadia Diamond-Smith, Prabhat Lamichhane

**Affiliations:** a The Aga Khan University South-Central Asia, East Africa; b Centre for Global Child Health, The Hospital for Sick Children, Toronto, Canada; c Department of Epidemiology and Biostatistics, Institute for Global Health Sciences, University of California, San Francisco, USA; d Department of Public Health, School of Psychology and Public Health, La Trobe University, Melbourne, Victoria, Australia

The World Health Organization defines preconception care as the provision of biomedical, behavioral, and social health interventions to women and couples before conception occurs, aiming to improve their health status and reduce factors contributing to poor maternal and child health outcomes.^1^ Others have suggested an even broader paradigm inclusive of societal needs.^2^ Although preconception care has been under the spotlight in Low- and Middle-Income countries (LMICs) for over a decade, corresponding progress in implementation and program development has been limited. Preconception nutrition issues such as undernutrition and concomitant overweight/obesity and micronutrient deficiencies, the so-called “triple-burden” of malnutrition, have received scant policy attention.

In this set of four Reviews,^3^, ^4^, ^5^, ^6^ authors from UNICEF South Asia and academic partners in UK and Australia explore aspects of preconception nutrition in the region based on local evidence, program and policy milieu and evidence gaps. Saville et al.^3^ review the evidence from preconception nutrition interventions in South Asia and find the strongest evidence for interventions that include food supplements, with or without multiple micronutrients. They suggest that multi-component interventions, while intensive, hold the most potential for impact. Miller et al.^4^ review a variety of data sources to understand the association between several indicators for preconception nutrition status (underweight, overweight, anaemia and micronutrient deficiencies) and health and socio-economic outcomes, finding mixed results. They argue that the lack of longitudinal studies on this population in South Asia poses challenges to understanding these complex relationships, a point also underscored by a paper on priority gaps on research related to preconception care^5^ in South Asia. Hazra et al.^6^ review existing programs and the policy milieu in South Asia to make the case for addressing bottlenecks, as well as developing and implementing comprehensive nutrition programs to address the unique needs for women. The dominant conclusions from the miniseries, supported by a parallel review of adolescent nutrition in South Asia,^7^ are that the entire area of optimizing maternal nutrition and wellbeing prior to pregnancy and childbirth is ripe for concerted action at regional, national and local level by policy makers and health care professionals.

What are the implications of these findings in relation to preconception nutrition in South Asia? With low fertility, young age at marriage, high reliance on sterilization after relatively short progressions from marriage to first to second birth, this concerns mostly relatively young and newly married women in South Asia. In the last National Family Health Survey in India,^8^ the median age at marriage among women 25–29 was 19.2 years and 21.2 years at the first birth. Over 60% of women became pregnant within 1 year of marriage.^9^ Due to a continued predominance of arranged or semi-arranged marriages, co-residence with in-laws, and patriarchal norms, young, newly married, preconception women are often at the lowest status in their households.^10^ This gives them little decision-making power, including about what, when, and how much they eat, and low autonomy to negotiate taking supplements.^11^ Additionally, this is exacerbated by cultural practices of young, newly married women eating last in their household. Thus, the women in most need of preconception nutrition support are often the most marginalized in their households and communities and are most at risk of already having low nutritional status.

Globally, as well as in South Asia, there is a need to scale up programs to address the triple-burden of malnutrition in all its forms and across the life course. Addressing the antecedents of poor nutrition in childhood and school age children is an essential forerunner to adequate nutrition in adolescence and youth to make a healthy transition into adult life. This will require leveraging the existing adolescent health and nutrition interventions and maternal programs while also introducing new interventions targeting the newly young married women. Given the largely patriarchal South Asian society, it is particularly important to engage civic society, increase awareness through mass media and targeted use of social media platforms. An important precursor here is engaging men and household members (parents and in-laws) to create a favorable environment for timing and desirability of pregnancies. Perhaps a starting point could be developing programs to optimize and measure health, nutrition and wellbeing for women across the life course, as a programmatic and policy continuum (12).

There are legitimate concerns that the terminology of preconception care incurs on reproductive rights and addresses the health and wellbeing of young women primarily through the lens of reproduction, which is inappropriate. Given the myriad health and wellbeing needs of adolescents and young women, there is a clear need to co-design holistic programs including reproductive health access (contraception) alongside nutrition across the entire continuum of childhood, adolescence and adulthood and nesting these upon the solid bedrock of social sector investments in education, poverty alleviation and social safety nets.

## Contributors

All authors contributed equally to the manuscript.

## Declaration of interests

Authors declare no conflict of interest.

## References

[1] World Health Organization (2013). https://iris.who.int/handle/10665/205637.

[2] Aynalem Y.A., Paul P., Olson J., Lassi Z.S., Meherali S. (2025). Preconception care: a concept analysis of an evolving paradigm. J Adv Nurs.

[3] Saville N.M., Dulal S., Miller F. (2025). Effects of preconception nutrition interventions on pregnancy and birth outcomes in south Asia: a systematic review. Lancet Reg Health Southeast Asia.

[4] Miller F., Sethi V., Schoenaker D. (2025). Preconception malnutrition among women and girls in south Asia: prevalence, determinants, and association with pregnancy and birth outcomes. Lancet Reg Health Southeast Asia.

[5] Miller F., Sethi V., Hazra A. (2025). Bridging the gaps: advancing preconception nutrition in south Asia through evidence, policy, and action. Lancet Reg Health Southeast Asia.

[6] Hazra A., Choedon T., Shrivastav M. (2025). Policies and programmes to improve preconception nutrition in South Asia. Lancet Reg Health Southeast Asia.

[7] Bhutta Z.A., Sharma D., Shafique S., Rashidi K. (2025). Improving adolescent health and nutrition in South Asia. BMJ.

[8] National family health survey NFHS-5, 2019-2021. https://mohfw.gov.in/sites/default/files/NFHS-5_Phase-II_0.pdf.

[9] Khalil U., Mookerjee S. (2019). Patrilocal status and women's social status: evidence from South Asia. Econ Dev Soc Change.

[10] Diamond-Smith N., Mitchell A., Cornell A. (2022). The development and feasibility of a group-based household-level intervention to improve preconception nutrition in Nawalparasi district of Nepal. BMC Public Health.

[11] Martopullo I., Neves P.A., Baird S. (2024). Understanding progress and challenges in women's health and wellbeing in exemplar countries: a time-series study identifying positive outliers. Lancet Glob Health.

